# Analysis of the Mechanisms Underlying the Specificity of the Variation Potential Induced by Different Stimuli

**DOI:** 10.3390/plants13202896

**Published:** 2024-10-16

**Authors:** Maxim Mudrilov, Maria Ladeynova, Yana Vetrova, Vladimir Vodeneev

**Affiliations:** Department of Biophysics, National Research Lobachevsky State University of Nizhny Novgorod, 23 Gagarin Avenue, 603022 Nizhny Novgorod, Russia

**Keywords:** abiotic stress, signal transduction, electrical signal, variation potential, hydraulic signal

## Abstract

Plants are able to perceive diverse environmental factors and form an appropriate systemic functional response. Systemic responses are induced by stimulus-specific long-distance signals that carry information about the stimulus. Variation potential is proposed as a candidate for the role of such a signal. Here, we focus on the mechanisms that determine the specificity of the variation potential under the action of different local stimuli. Local stimuli such as heating, burning and wounding cause variation potential, the parameters of which differ depending on the type of stimulus. It was found that the stimulus-specific features of the hydraulic signal monitored by changes in leaf thickness and variation potential, such as a greater amplitude upon heating and burning and a significant amplitude decrement upon burning and wounding, were similar. The main features of these signals are the greater amplitude upon heating and burning, and a significant amplitude decrement upon burning and wounding. Together with the temporal correspondence of signal propagation, this evidence indicates a role for the hydraulic signal in the induction of stimulus-specific variation potential. Experiments using mechanosensitive channel inhibitors have demonstrated that the hydraulic signal contributes more to the induction of the variation potential in the case of rapidly growing stimuli, such as burning and wounding, than in the case of gradual heating. For thermal stimuli (gradual heating and burning), a greater contribution, compared to wounding, of the chemical signal related to reactive oxygen species to the induction of the variation potential was demonstrated. Thus, the specificity of the parameters of the variation potential is determined by the different contributions of hydraulic and chemical signals.

## 1. Introduction

To date, sufficient evidence has accumulated that local stimuli trigger systemic functional responses that cover the whole plant body [1,2]. Such responses are known to be stimulus-specific and involve unstimulated parts of the plant. First of all, it is worth noting the stimulus-specific differences in the dynamics of various phytohormones [3,4,5,6,7] and metabolites [4,8], the dynamics of photosynthesis and transpiration responses [3,4,5,6], the level of expression of various genes [6,7,8,9], etc. Such stimulus-specific systemic responses can only be induced by a stimulus-specific long-distance signal that carries information about the stimulus.

One such signal may be the variation potential (VP), which is a transient depolarization of irregular shape and duration [1,2,10,11,12]. VP occurs in response to various damaging stimuli [1,2,10,11], and its parameters, such as the amplitude and propagation velocity, may depend on the type of stimulus [5,13] and the area of damage [14]. Experiments on the stimulus specificity of the VP parameters revealed that in the case of rapidly growing stimuli, such as burning and wounding, there is a significant decrement during the VP propagation, whereas in the case of slowly growing stimuli, such as gradual heating, there is almost no decrement [5,13]. Most importantly, it was shown that VP can induce a stimulus-specific systemic response [1,2]. Thus, VP can potentially regulate the systemic response by changing its parameters. 

However, the outstanding question remains: how are VP parameters regulated upon different stimulations? The specificity of VP may be based on the features of its mechanisms of generation and propagation, due to its complex nature: VP is an electrical reaction in response to a hydraulic or chemical signal, or a combination of both [1,2,11,12]. The role of the hydraulic signal is evidenced by data on the induction of VP by artificially increasing intra-vessel fluid pressure [14,15], data on increases in the thickness of leaves or stems preceding the VP [16,17], as well as good agreement with the results of mathematical ing [18,19]. It is assumed that chemical signals may be some wounding substances propagating from the damage site throughout vascular bundles, such as reactive oxygen species (ROS) produced by NADPH oxidases [2,11,20,21,22], which is supported by the similar dynamics of ROS wave propagation [11,21]. It is proposed that hydraulic and chemical signals activate mechanosensitive and/or ligand-gated Ca^2+^-permeable channels, respectively, inducing an initial step of membrane potential changes during VP generation [2,11]. An important role in the formation of the specificity of VP parameters can be played by the physical features of the propagation of hydraulic and chemical signals, for example, the higher propagation velocity of hydraulic signals compared to chemical ones [11]. This can lead to the activation of Ca^2+^-permeable channels of different types and different lag times for their activation, which determines the characteristics of the VP parameters. Thus, differences in the parameters of the VPs caused by various stimuli may be primarily due to the different contributions of chemical and hydraulic signals to the induction of the VP.

However, the precise mechanism of the specificity of VP parameters needs investigation. In summary, the present work aimed to identify the stimulus-specific features of the mechanisms of generation and propagation of VP in wheat plants.

## 2. Results

### 2.1. Parameters of Variation Potentials Induced by Different Local Stimuli

**Figure 1:**
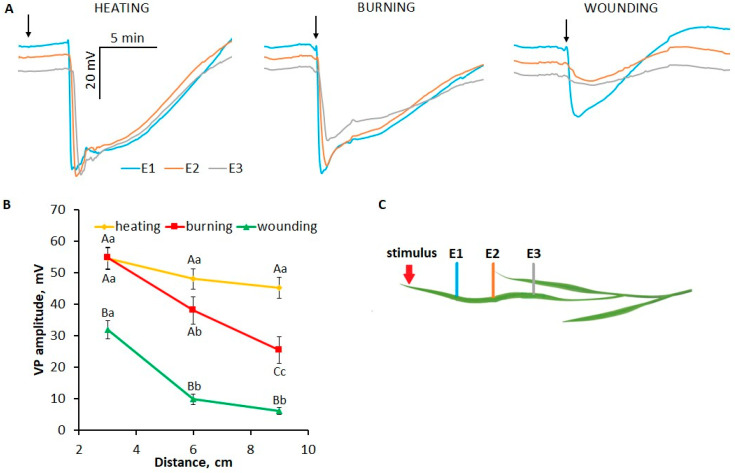
Variation potentials (VPs) induced by local heating, burning or wounding in wheat plants. (**A**) Averaged VP traces. The arrow indicates the moment of mechanical wounding, burning or the beginning of gradual heating of the leaf tip. (**B**) Dependence of the VP amplitude on the distance to the area of local stimulation. Data are means ± SEM. Different uppercase letters indicate statistically significant differences between stimuli; different lowercase letters indicate statistically significant differences between distances within a single stimulus (*p* < 0.05). (**C**) Schematic representation of the experimental design for monitoring surface potentials in wheat plants. E1, E2, E3: surface electrodes.

Heating, burning and mechanical wounding of the tip of a wheat leaf caused the generation of a long-distance electrical signal in the form of a transient depolarization of irregular shape and duration (Figure 1A), with characteristics corresponding to VP [1,2,11]. In the cases of heating and burning, the VP amplitudes near the site of stimulation (3 cm) were similar (~54.5 ± 3.5 mV), whereas in the case of wounding it was significantly lower (~32 ± 3 mV). As VP propagates, its amplitude decreases; the degree of attenuation depends on the type of stimulus. In cases of burning and wounding, the VP amplitude was significantly attenuated: at a distance of 9 cm from the stimulation site, the amplitude was 46% and 19% of the initial amplitude (at 3 cm), respectively. By contrast, the amplitude of the heat-induced VP was attenuated to 83% of the initial amplitude (Figure 1B). These results demonstrate the dependence of VP parameters, such as amplitude and decrement, on the type of stimulus. These findings suggest that there are stimulus-specific differences in the mechanisms of VP generation and propagation.

### 2.2. The Role of the Hydraulic Wave in the Propagation of Variation Potentials Caused by Different Local Stimuli

**Figure 2:**
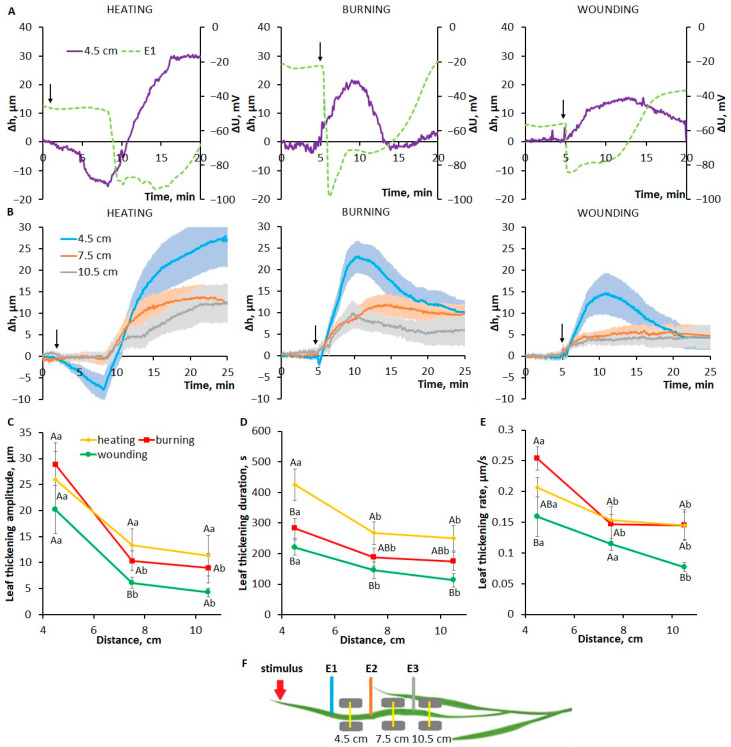
Systemic changes in leaf thickness induced by local heating, burning or wounding in wheat plants. (**A**) Simultaneous representative recordings of leaf thickness changes (Δh, solid lines) and variation potentials (ΔU, dashed lines) at a distance of 4.5 cm and 3 cm, respectively, from the stimulation area. The arrow indicates the moment of mechanical wounding, burning or the beginning of gradual heating of the leaf tip. (**B**) Averaged recordings of leaf thickness changes (Δh); shaded regions (envelopes) represent SEM. The arrow indicates the moment of mechanical wounding, burning or the beginning of gradual heating of the leaf tip. (**C**–**E**) Dependences of the amplitude (**C**), duration (**D**) and rate (**E**) of leaf thickening on the distance to the area of local stimulation. Data are means ± SEM. Different uppercase letters indicate statistically significant differences between stimuli; different lowercase letters indicate statistically significant differences between distances within a single stimulus (*p* < 0.05). (**F**) Schematic representation of the experimental design for simultaneous monitoring of surface potentials and changes in wheat leaf thickness. E1, E2, E3: surface electrodes. Yellow lines are light bands between the sender units and the receiver units of the optical micrometers (gray rectangles).

Heating, burning and mechanical wounding induced a hydraulic signal that caused an increase in leaf thickness (Figure 2B). The main stimulus-specific features of changes in leaf thickness at a distance of 4.5 cm from the stimulation area will be discussed below. A slight decrease in leaf thickness preceded leaf thickening (Figure 2A,B and Figure S1). In cases of burning and wounding, the amplitude of the thickness reduction phase was small (<5 µm), and its duration did not exceed several seconds. In the case of heating, the amplitude of the thickness reduction phase was several times greater than that upon burning and wounding, and its duration was several minutes (Figure S1).

The amplitude (Figure 2C) and duration (Figure 2D) of the phase of increasing leaf thickness were significantly greater compared to the thickness reduction phase. The amplitude of thickening upon wounding was about 15–20 µm, whereas upon heating and burning it reached 20–30 µm. The duration of the thickening phase, i.e., the time taken from the beginning of an increase in leaf thickness until the thickness reached its maximum, was greatest upon heating compared to other stimuli (Figure 2D). The leaf thickening rate was lowest upon wounding, higher upon heating and highest upon burning (Figure 2E).

It is important to note that the thickening phase began almost simultaneously with the VP generation under the action of stimuli of all types (Figure 2A), suggesting that the hydraulic signal is essential for VP induction.

The thickness change parameters show a dependence on the distance from the stimulation area. The amplitude of the heat-induced increase in leaf thickness decreased slightly with increasing distance from the stimulation site, whereas it greatly decreased in the case of burning and, especially, in the case of wounding (Figure 2C). The ratios of the durations of the thickening phases between stimuli were maintained as the distance from the stimulation area increased (Figure 2D). Thus, hydraulic-signal-induced changes in leaf thickness also depend on the stimulus type in a similar manner to VP parameters.

### 2.3. Systemic Changes in Stomatal Conductance Induced by Different Local Stimuli

**Figure 3:**
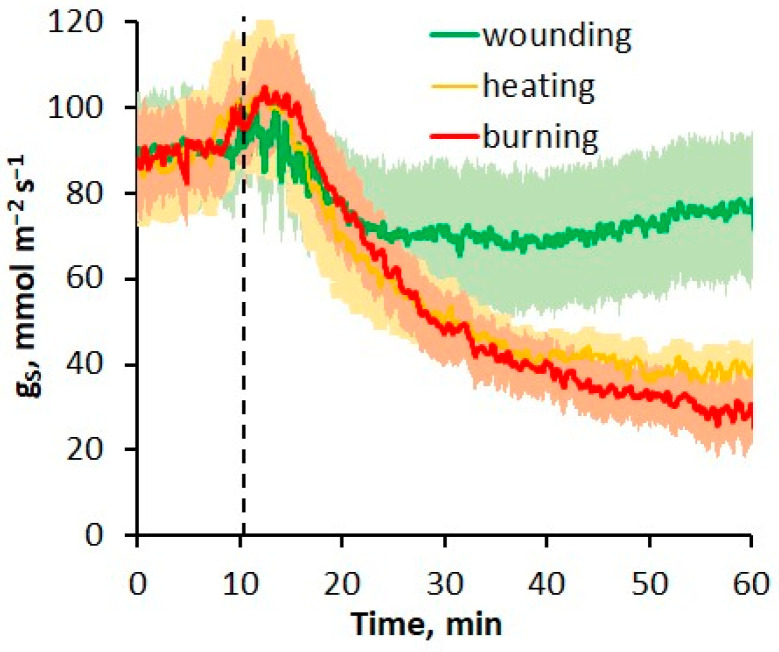
The dynamics of stomatal conductance (g_S_) induced by local heating, burning or wounding in the unstimulated part of the wheat plant. Lines are means; shaded regions (envelopes) represent SEM. The dashed line indicates the moment of generation of the variation potential.

The observed increase in wheat leaf thickness due to the propagation of the hydraulic signal may be associated with changes in water exchange. One of the important components of water exchange is water loss through the stomata. Changes in stomatal conductance (g_S_) induced by local heating, burning or mechanical wounding were investigated in the unstimulated part of the wheat leaf. Changes in g_S_ were biphasic, with an initial slight increase in transpiration rate followed by a significant decrease (Figure 3). Note that the first phase of increasing the transpiration rate either began simultaneously with the VP generation or preceded it by several seconds. The phase of the decrease in transpiration rate was more pronounced and prolonged. The amplitude of g_S_ changes was the smallest upon wounding (g_S_ decrease by 1/4 from the resting level), whereas it was significantly greater upon burning and heating.

### 2.4. Parameters of Variation Potential and Hydraulic Signal in a Detached Wheat Leaf

**Figure 4:**
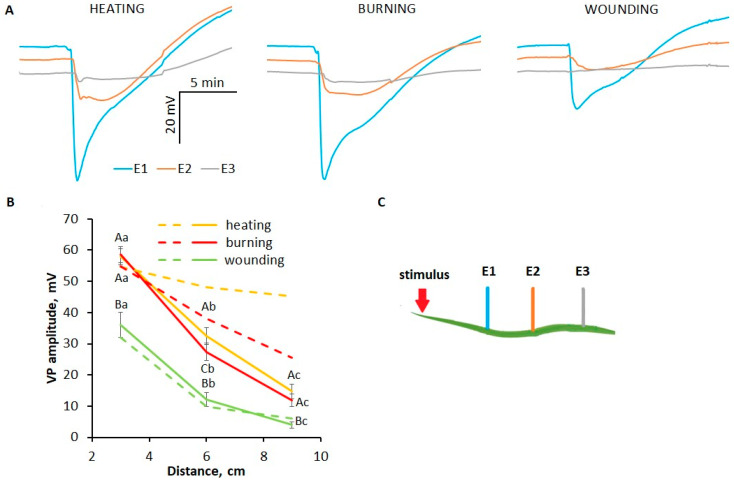
Variation potentials (VPs) induced by local heating, burning or wounding in the detached wheat leaf. (**A**) Averaged VP traces. The arrow indicates the moment of mechanical wounding, burning or the beginning of gradual heating of the leaf tip. (**B**) Comparison of the dependences of the VP amplitude on the distance to the area of local stimulation in detached leaves (solid lines) and whole wheat plants (dashed lines). Data are means ± SEM. For detached leaves only, different uppercase letters indicate statistically significant differences between stimuli; different lowercase letters indicate statistically significant differences between distances within a single stimulus (*p* < 0.05). (**C**) Schematic representation of the experimental design for monitoring surface potentials in wheat plants. E1, E2, E3: surface electrodes.

The study of stimulus-specific features of the mechanisms of propagation and generation of VP was performed on the basis of inhibitor analysis. Due to the low penetration of inhibitors through the wheat leaf epidermis, experiments with inhibitors were carried out using a detached wheat leaf. To assess the suitability of the detached leaf model, VP parameters in whole plants and detached leaves were compared. The VP in the detached leaves had its own characteristic shape (Figure 4A). VP amplitudes near the stimulation area (3 cm) in whole plants and excised leaves were very similar. However, the VP propagation in the detached leaf was altered: VP amplitude was more attenuated with increasing distance from the stimulation site. The differences were more pronounced in the case of heating. At distances of 6 cm and 9 cm, the amplitudes were reduced to 56% and 25% of the initial amplitude (at 3 cm), respectively (Figure 4B), whereas a decrement was almost not observed in whole plants (Figure 1B). The decrease in amplitude was 47% and 11% at a distance of 6 cm and 34% and 11% at a distance of 9 cm from the initial amplitude upon burning and wounding, respectively (Figure 4B). Notwithstanding, the main stimulus-specific differences between VP parameters were clearly identified using detached leaves as in whole plants.

**Figure 5:**
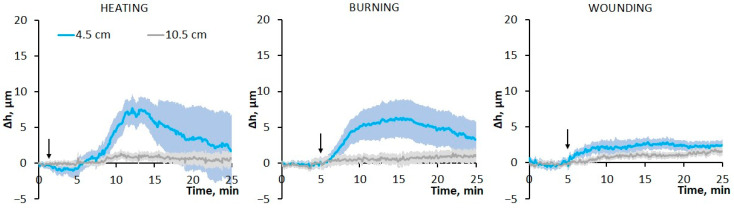
Systemic changes in leaf thickness (Δh) induced by local heating, burning or wounding in the detached wheat leaf. Lines are means; shaded regions (envelopes) represent SEM. The arrow indicates the moment of mechanical wounding, burning or the beginning of gradual heating of the leaf tip.

The effect of leaf detachment on hydraulic signal parameters was also analyzed (Figure 5). A significant decrease in leaf thickening amplitude was found for all stimulus types, both near the stimulation site (4.5 cm) and at a distance of 10.5 cm. However, as for VP, the main stimulus-specific features of the hydraulic signals were also clearly identified in detached leaves, such as the presence of a pronounced and prolonged phase of thickness reduction upon heating, as well as the greatest amplitude of the thickening phase in the case of heating and the smallest in the case of wounding. It can also be noted that the changes in hydraulic signals caused by the leaf detachment were similar to those for the VP.

Similarities in the dependences of the parameters of VP and hydraulic signal on the type of stimulus between whole plants and detached leaves indicate the suitability of the detached leaf model for studying the mechanisms of VP generation and propagation.

### 2.5. Contribution of Reactive Oxygen Species to the Propagation of Variation Potentials Caused by Different Local Stimuli

**Figure 6:**
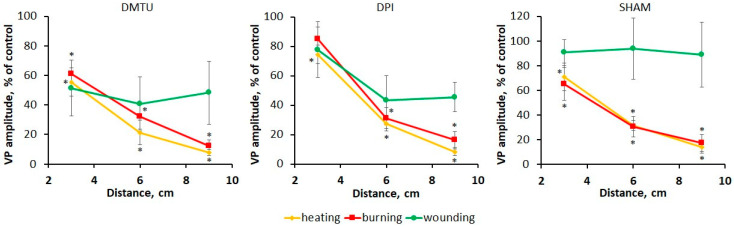
Effects of the reactive oxygen species (ROS) scavenger N,N′-dimethylthiourea (DMTU) and ROS-producing enzyme inhibitors diphenyleneiodonium chloride (DPI) and salicylhydroxamic acid (SHAM) on the amplitudes of the variation potentials (VPs) induced by local heating, burning or wounding in the detached wheat leaf. VP amplitude is represented as the percentage of control, which is the VP amplitude in untreated leaves (without scavengers and inhibitors). Data are means ± SEM. * indicates data significantly different from untreated leaves (*p* < 0.05).

In addition to the hydraulic signal, the VP propagation is associated with a chemical signal, the role of which, according to hypotheses in some works [2,11,22], can be played by ROS. The possible contribution of ROS to the VP propagation was evaluated using an ROS scavenger N,N′-dimethylthiourea (DMTU) [23,24]. Treatment with DMTU led to a decrease in the VP amplitude near the site of stimulation (3 cm) under the action of all stimuli (Figure 6). VP propagation was more suppressed upon heating compared to burning and wounding. To determine the ROS sources, the inhibitor of NADPH oxidases diphenyleneiodonium chloride (DPI) [25,26] and salicylhydroxamic acid (SHAM), which inhibits cell wall peroxidases [27] and mitochondrial alternative oxidases [28], were used. These inhibitors slightly reduced the VP amplitude near the stimulation area but significantly suppressed the propagation of heat- and burn-induced VPs. The propagation of the wound-induced VP was less inhibited, especially upon SHAM treatment. The inhibition of ROS production also resulted in a change in VP amplitude decrement, which was more than 75% at a distance of 6 cm from the stimulation area for all stimulus types except SHAM treatment upon wounding. These results suggest that DPI- and SHAM-inhibited oxidases are involved in maintaining the propagation of the heat- and burn-induced VPs through active ROS production [2], whereas they are probably not involved in the propagation of the wound-induced VP.

Thus, the different contributions of ROS indicate, first of all, stimulus-specific differences in the parameters of VP propagation, but not differences in the VP amplitude near the stimulation area. Therefore, further studies are needed to determine the stimulus-specific features of the VP generation mechanism.

### 2.6. Analysis of the Features of the Mechanisms of Generation of Variation Potentials Induced by Different Local Stimuli

**Figure 7:**
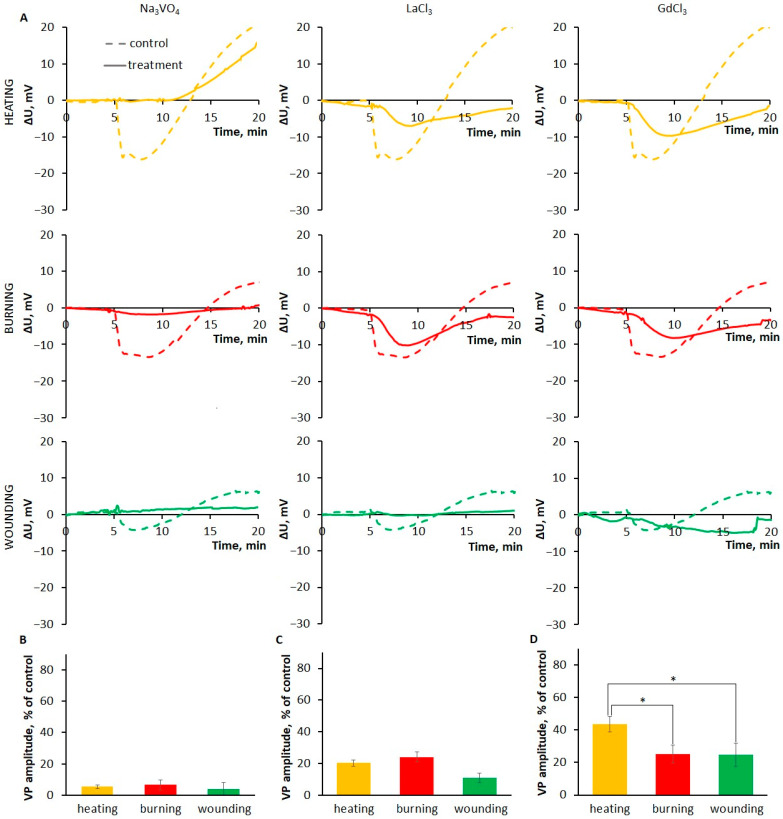
Effects of the H^+^-ATPase inhibitor Na_3_VO_4_, the Са^2+^-permeable channel blocker LaCl_3_ and the mechanosensitive channel inhibitor GdCl_3_ on the variation potentials (VPs) induced by local heating, burning or wounding in the detached wheat leaf. (**A**) Averaged VP traces observed at a distance of 6 cm from the stimulation area in untreated leaves (dashed lines) or those treated with the inhibitor/blocker (solid lines). (**B**–**D**) VP amplitude at a distance of 6 cm from the area of local stimulation upon treatment with Na_3_VO_4_ (**B**), LaCl_3_ (**C**) or GdCl_3_ (**D**). VP amplitude is represented as the percentage of control, which is the VP amplitude in untreated leaves (without inhibitor). Data are means ± SEM. Statistically significant differences between untreated and treated leaves were found for all stimuli. * indicates significant differences between the stimuli (*p* < 0.05).

To study the features of the VP generation mechanism, an inhibitor analysis using inhibitors of ion transport systems was used. It is known that H^+^-ATPase inactivation plays a key role in VP generation [2,10,11,22]. Treatment with an H^+^-ATPase inhibitor sodium orthovanadate resulted in the almost complete suppression of the VP for all types of stimuli; the VP amplitude was less than 10% of the amplitude in untreated leaves (Figure 7B).

Changes in H^+^-ATPase activity may be related to an increase in cytosolic Ca^2+^ concentration due to the activation of Ca^2+^-permeable channels [2,29]. It is proposed that both mechanosensitive and ROS-activated Ca^2+^-permeable plasma membrane channels are involved in VP generation [2,11,22,29]. 

Treatment with the Ca^2+^-permeable plasma membrane channel blocker LaCl_3_ led to a significant (more than 50%) decrease in VP amplitude for all stimuli (Figure 7C). It should be noted that, along with the suppression of amplitude, there was a pronounced decrease in the rate of depolarization (Figure 7A).

To analyze the involvement of mechanosensitive channels in the generation of VPs induced by different stimuli, the effects of the inhibitor GdCl_3_ on the VP parameters were evaluated. It was shown that treatment with the GdCl_3_ led to a decrease in VP amplitude and depolarization rate for all stimuli, but a greater effect was observed in the cases of burning and wounding, which was expressed as a decrease in amplitude by more than 60% compared to untreated leaves (Figure 7D).

Thus, a greater contribution of mechanosensitive channels to VP generation was revealed in the cases of burning and wounding compared to gradual heating.

## 3. Discussion

The analysis of the parameters of VPs induced by different local stress stimuli revealed stimulus-specific differences. The smallest amplitude was in the case of wounding, which rapidly attenuated during VP propagation, and the greatest amplitude was near the stimulation area in the cases of heating and burning, which had a pronounced decrement upon burning and a small decrement upon heating (Figure 1). The stimulus-specific features of VPs in wheat plants revealed in this work correspond to those in pea plants [13].

As noted, VP is not a self-propagating signal but is an electrical reaction that is induced by a hydraulic or chemical signal, or a combination of both [1,2,11,12]. It can be proposed that the stimulus-specific features of the electrical reaction at a distance from the stimulation zone are due to the features of these signals, which are discussed below.

### 3.1. The Role of the Hydraulic Signal in the Formation of the Specificity of Variation Potentials Induced by Different Local Stimuli

To study the role of the hydraulic signal in VP induction, an analysis of systemic changes in leaf thickness in response to different stimuli was performed. The stimulus-induced increase in leaf thickness was several micrometers, lasted for tens of minutes and was preceded by a decrease in leaf thickness of shorter duration and smaller amplitude (Figure 2). Hydraulic signals, monitored by changes in the thickness of leaves or stems or other methods, have been shown in previous works in response to externally applied pressure [15], burning [15,16,30,31,32], insect feeding [17,30], and mechanical wounding [17,33]. The thickness change parameters from previous studies are in good agreement with the data in this work: a duration of up to tens of minutes, an amplitude of several micrometers [15,16,17,30,31], and a decrement in amplitude with distance from the stimulation area [16,31]. Stimulus-specific differences in the parameters of changes in leaf thickness have also been shown previously, in particular between burning and insect feeding [30].

This study demonstrated that the stimulus-specific features of VP and leaf thickening parameters were similar (Figure 1 and Figure 2), suggesting a hydraulic signal as a trigger for both leaf thickening and VP. This was also evidenced by the fact that an increase in leaf thickness preceded or coincided with VP generation (Figure 2A) [16,17,32]. VP occurred when the increase in leaf thickness was at the initial stage (Figure 2A), which may indicate a small threshold for VP generation.

The mechanisms of VP induction and the increase in leaf thickness by a hydraulic signal are open questions. Pressure wave propagation or xylem mass flow may be these mechanisms [11,17]. Hydraulic pressure waves propagate in liquid at very high speeds and can cause rapid changes in the size of vessels, which causes deformations of adjacent parenchyma cells [11,19,34]. In the case of hydraulic mass flow, there is a direct fluid translocation through the vessels, propagating at a much lower speed, but also capable of causing a change in both the size of the vessels and the cells surrounding them [11,18,35]. These changes may lead to an increase in leaf thickness [14,18,19] and to VP induction through the activation of mechanosensitive ion channels in the plasma membrane of parenchyma cells adjacent to the xylem [2,11,19]. These channels are activated in response to changes in plasma membrane tension (including sensing membrane tension directed from the outside of the cell), as well as in response to cell turgor changes [36,37] caused by water movement from the xylem during hydraulic mass flow.

First, the role of the hydraulic pressure wave as a possible VP inducer will be discussed. A wave of positive pressure changes originates as a result of wound-induced damage to xylem vessels and the subsequent release of xylem water column tension [11], since under normal conditions, there is negative pressure in the xylem as a result of transpiration [38,39]. This can potentially cause leaf thickness increase and VP generation [32], which is supported by data on the possibility of inducing tissue deformation and VP-like electrical signals by artificially increasing the pressure in the xylem [14,15,40]. Moreover, the amplitudes of VP-like electrical signals depend on the size of the applied pressure steps [14]. The role of the hydraulic pressure wave in VP induction is supported by the dependence of the VP amplitude on the values of the initial negative pressure (tension) in the xylem [14,40], as well as by the fact that VP is not observed when the initial xylem pressure is positive [14,32].

However, there are some contradictory facts regarding the involvement of the hydraulic pressure wave in the VP induction mechanism. First, although there is some flexibility in the cell walls of xylem vessels [41,42,43], it is unlikely that, under normal conditions, pressure-induced changes in xylem vessel size will be sufficient to cause significant changes in leaf thickness. Moreover, even under severe drought, significant deformations of xylem vessels cause changes in cell diameter not exceeding a few micrometers [41,43]. Secondly, xylem pressure is unlikely to change significantly with increasing distance from the stimulation site, even taking into account the gradient of negative xylem pressure from root to shoot [14,40,44]. Thirdly, due to the high propagation speeds of the pressure wave in the liquid [11,17,34], the observed changes in leaf thickness (Figure 2) should occur almost instantaneously; however, there is a time lag between the moment of stimulation and the phase of increasing leaf thickness.

Another potential mechanism for increasing leaf thickness and VP induction is xylem mass flow, which results in the movement of fluid from the xylem to surrounding cells and an increase in their turgor pressure. An increase in the volume of many parenchyma cells can lead to a significant increase in leaf thickness. In addition, there is evidence of the possibility of VP induction by changing the turgor of the cells surrounding the xylem [14]. Moreover, one study showed that water influx was required for VP propagation upon stem excision [45], i.e., pressure change alone was insufficient to trigger VP, suggesting the involvement of hydraulic mass flow. Finally, hydraulic mass flow can facilitate the propagation of chemical signals that cause the activation of ligand-gated ion channels and VP induction [1,2,18], which is supported by the results of this study (Figure 6). Taken together, the evidence described above is more consistent with the hypothesis of hydraulic mass flow as the main VP inducer.

The next question is about the water supply involved in hydraulic mass flow. The most likely supply of water to the xylem is from the apoplast and damaged cells of the wounded area [16,30,46]. In one study, calculations showed that wound-induced changes in leaf thickness depended on the amount of water available in the stimulation area [30]. It should also be noted that increasing positive pressure in the damage site can promote hydraulic mass flow, as evidenced by the increased xylem flow rate upon wounding [44,47].

In addition to positive pressure changes due to the damage-induced release of xylem tension, water supply may be facilitated by the inhibition of transpiration, which under normal conditions provides an upward flow of fluid [35,39,44]. It is well known that stimuli such as heating [5], burning [5,48] and mechanical damage [49] cause a significant decrease in stomatal conductance and transpiration, which was also shown in this work (Figure 3). This leads to a positive change in xylem pressure and disruption of the normal fluid flow in the xylem, which probably underlies the dependence of the amplitude and velocity of VP propagation on the transpiration rate [50].

Next, possible mechanisms of stimulus-specific features of the parameters of leaf thickness changes will be discussed. In the case of heating and burning, the greater amplitude of leaf thickening compared to wounding (Figure 2B) could have been caused by heat-driven water expansion [14,17]. In the case of burning, this process was probably faster and more severe due to the higher flame temperature, providing a high rate of increase in leaf thickness compared to heating (Figure 2E). The longer duration of the leaf thickening phase upon heating was likely due to the longer duration of stimulation (Figure 2D), leading to an increased amount of water available for hydraulic mass flow (Figure 2C).

Another factor that determines the greater amplitude and duration of leaf thickening in the case of heating may be the large contribution of changes in transpiration, since leaf thickness reached its maximum 10–15 min after stimulation (Figure 2B), when the transpiration rate decreased significantly (Figure 3). In the case of burning and wounding, the influence of transpiration on changes in leaf thickness was most likely insignificant, since the decrease in stomatal conductance began no earlier than 5 min after stimulation (Figure 3), when the increase in leaf thickness had reached its maximum (Figure 2B).

The rather large decrease in leaf thickness observed during heating, which preceded the VP generation (Figure 2A), can be explained by an increase in the transpiration rate (Figure 3), which led to a tension increase in the xylem sap column (negative pressure) [39,40,44,51] and, as a consequence, to an accelerated efflux of water from systemic tissues to the heated area. The increase in the transpiration rate may be associated with both an increase in temperature [6,52] and with the hydropassive opening of stomata, possibly due to the loss of turgor in the epidermis [5,48].

The role of hydraulic mass flow in VP induction is also supported by the results of experiments on detached leaves, which showed a significant reduction in VP amplitude and an increase in decrement, particularly in the case of heating (Figure 4B). This suggestion is based on the fact that the pressure in the xylem vessels of a detached leaf is more positive compared to the whole plant due to a loss of vessel integrity upon excision [14], which leads to a reduced damage-induced pressure drop, and as a consequence, to impaired hydraulic mass flow and VP propagation. Another reason for the suppression of hydraulic mass flow and VP may be the influence of other plant parts, potentially through two ways: first, by changing the total amount of water available for hydraulic mass flow [30], and second, by altering xylem water column tension (more negative pressure in intact plants due to xylem tension in other leaves and active transpiration) [39,40,51].

### 3.2. The Role of the Chemical Signal in the Formation of the Specificity of Variation Potentials Induced by Different Local Stimuli

Along with the hydraulic signal, the chemical signal is proposed as a VP inducer, the role of which can be played by ROS [2,11,20,21,22]. To determine the contribution of ROS to the VP propagation caused by different stimuli, the effects of ROS scavengers and ROS production inhibitors on VP amplitude were evaluated. Treatment with DMTU led to a suppression of VPs, induced by all stimuli (Figure 6). VP suppression was more pronounced at a distance from the stimulation area, supporting the role of ROS in VP propagation. The suppression of the wound-stimulated VP was the smallest, suggesting a smaller contribution of ROS to the induction of wound-stimulated VP compared to other stimuli. This appears to be related to a reduced initial ROS burst in the stimulated area compared to burning and heating due to the lack of thermal exposure [53,54,55]. 

In addition to ROS transported by xylem mass flow from the stimulation area, the systemic production of ROS is possible, maintaining the propagation of the ROS wave in unstimulated parts of the plant [2,11,56,57,58]. These propagating ROS signals appear to be dependent on ROS-producing enzymes, which are inhibited by DPI [25,26] and SHAM [27,28]. This is supported by the significant VP suppression by these inhibitors at a distance from the stimulation site (Figure 6). According to the literature, the central role in the systemic propagation of ROS waves is played by DPI-inhibited NADPH oxidases, most likely RESPIRATORY BURST OXIDASE HOMOLOGUE D (RBOHD) [8,20,54,57,59]. This is consistent with the results that VPs were more attenuated upon DPI treatment compared to SHAM treatment (Figure 6). Moreover, the results of this work are in good agreement with data from other studies in which VP propagation in systemic tissues was suppressed in loss-of-function *rbohD* mutants, but local membrane potential changes were not suppressed in the *rbohD* mutant [8,33]. 

It should be noted that the statistically significant VP suppression by inhibitors of ROS production compared to control treatment was observed only upon heating and burning, but not upon wounding (Figure 6). In addition to the aforementioned reduced initial ROS burst, the more attenuated ROS wave upon wounding was possibly due to lower systemic ROS production, which could be related to the different mechanisms of occurrence of this wave upon different stimulations. This is supported by the fact that the regulatory mechanisms and propagation pathways of the ROS wave in Arabidopsis are different in response to the local application of high-light stress on the one hand, and in response to wounding [33] and heating [6] on the other hand, while the function of RBOHD is required in response to all stimuli.

### 3.3. The Features of Generation of Variation Potentials Induced by Different Stimuli

Inhibitor studies have revealed the universality of the involvement of H^+^-ATPase in the generation of VPs caused by different local stimuli. H^+^-ATPase is the major contributor to VP generation (Figure 7). However, it should be noted that the suppression of VPs during inhibition of H^+^-ATPase may be associated not only with its direct contribution to generation, but also with the dissipation of gradients of other ions [60,61].

The results of this work are consistent with other studies that have demonstrated the involvement of H^+^-ATPase in VP generation upon wounding [62,63], burning [5,60,64,65] and heating [5,66,67], including by detecting pH changes during VP generation [5,65,67]. It can be assumed that the absence of differences in VP parameters between stimuli upon inhibitor treatment (Figure 7) is due to a similar main contribution of H^+^-ATPase, probably H^+^-ATPase 1 (AHA1) [62,63], to the change in membrane potential during VP generation [2,10,11,22,29,56], although limitations of the approach used, inhibitor analysis, cannot be excluded.

The universality of the involvement of Ca^2+^-permeable plasma membrane channels in the generation of VPs induced by different local stimuli was also demonstrated (Figure 7). Other studies have previously shown the involvement of Ca^2+^-permeable channels in in VP generation upon heating [66] and burning [64,68], including studies using inhibitors of Ca^2+^-permeable channels [64,66] and Ca^2+^ chelators [64,66,68]. When Ca^2+^-permeable channels are inhibited, the VP amplitude is suppressed less than when H^+^-ATPase is inhibited (Figure 7), which can be associated with the aforementioned reduction in the electrochemical potential gradient [60,61].

Treatment with the mechanosensitive channel inhibitor GdCl_3_ led to the suppression of VPs caused by all stimuli (Figure 7), further indicating the involvement of hydraulic signals in the VP generation and propagation. Treatment with GdCl_3_, like treatment with LaCl_3_, led to a decrease in the rate of depolarization, which suggests that the mechanosensitive channels are involved in the formation of the rapid depolarization phase for all types of stimuli. These channels appeared to contribute more to the generation and propagation of burn- and wound-induced VPs than heat-induced VPs, as evidenced by a decrease in VP amplitude upon GdCl_3_ treatment (Figure 7). It is possible that this may be associated with the rate of pressure increase (slope) during the hydraulic signal, which is higher in the case of burning and, partly, wounding, since a dependence of activation on the rate of pressure increase has been demonstrated for mechanosensitive plant channels [69]. The results of this study suggest a greater contribution of mechanosensitive channels to the generation of VPs in the case of burning and wounding.

The molecular identities of the signaling components underlying the specificity of the mechanisms of VP generation and propagation, and the mechanisms of their interplay, are still not clear. ROS-activated Ca^2+^-permeable plasma membrane channels have been suggested to be involved in VP generation and propagation, but the molecular identity of these channels remains unknown [29,70,71,72]. To date, ligand-gated Ca^2+^-permeable cation channels of the GLUTAMATE RECEPTOR-LIKE (GLR) family have been confirmed to be essential to generating VPs [61,70]. Among the channels of the GLR family, the contribution to the VP generation and propagation was experimentally shown for GLR3.3 [33,54,63,73,74,75,76], GLR3.6 [33,54,63,73,74,75], GLR3.1 and GLR3.5 [74,75]. It should be noted that GLR3.3 and GLR3.6 are required for wound-induced systemic Ca^2+^ waves [63,74,76,77,78], hydraulic waves [33] and RBOHD-mediated ROS waves [33,54,79].

The initial perception of hydraulic waves may be mediated by the stretch-activated anion channel, MECHANOSENSITIVE CHANNEL OF SMALL CONDUCTANCE-LIKE 10 (MSL10), which plays a critical role in the proper formation of VPs through the regulation of GLR3.3 and GLR3.6 activity [80], which links this channels to hydraulic waves. It should be emphasized that for MSL10, the dependence of activation on the rate of pressure increase is shown [69]. It is possible that other mechanosensitive channels may also be involved in VP generation and propagation [61].

It should be noted that the above-mentioned GLR3.3 and GLR3.6 may also be involved in the interplay of various components of the complex plant stress signal—Ca^2+^, ROS and hydraulic waves—providing crosstalk between the signals. Thus, based on the similar suppression of wound-induced Ca^2+^ waves in the *glr3.3;glr3.6* double mutant and *rbohD* mutants, it can be assumed that RBOHD is involved in the regulation of Ca^2+^ signals [33,54], which in turn provides additional ROS production [11,58]. At the same time, it was shown that wound-induced GLR3.3-mediated calcium waves are RBOHD independent [77]. It has also been shown that GLR3.3 and GLR3.6 are involved in maintaining ROS waves induced by certain stimuli [33]. Thus, wound-induced ROS waves were suppressed in the *glr3.3;glr3.6* double mutant. At the same time, in response to high-light stress, ROS waves were not completely suppressed [33], indicating a different mechanism for ROS wave propagation [6,33] and, in general, the presence of the specificity of this signal caused by different stimuli.

It is important to note that no influence was found of the key ROS wave generator, RBOHD, on the other component of the complex stress signal, which is the hydraulic wave [33]. The mechanism of water transport into cells surrounding the xylem during hydraulic mass flow may involve aquaporins, such as plasma membrane intrinsic protein 2;1 (PIP2;1) [33,81]. Aquaporins are known to play a role in regulating the hydraulic conductance of vascular bundles [81]. The wound-induced systemic hydraulic signal is suppressed in the *pip2;1* mutant. The function of aquaporins is regulated by calcium, likely mediated by the cation channels GLR3.3 and GLR3.6. According to the authors, an increase in cytosolic Ca^2+^ concentration leads to the closure of aquaporins and a subsequent increase in hydraulic pressure in the xylem vessels due to the abolished efflux of water into the cells surrounding the xylem [33]. In addition, aquaporins such as PIP2;1, as well as plasmodesmata (PD)-localized proteins (PDLP) 1 and 5 play key roles in regulating rapid systemic ROS signals [33,82].

Thus, it can be proposed that the mechanisms of stimulus-specific VP induction and propagation are based on the different contributions of the hydraulic and chemical components of VP. Stimulus-induced damage results in the release of xylem water column tension and the release of chemical elicitors such as ROS and water from damaged cells. In the case of thermal stimuli, more ROS are produced compared to mechanical wounding due to thermal exposure. ROS are transported by xylem mass flow. During hydraulic mass flow, water moves into the cells surrounding the xylem, resulting in increased cell turgor and the subsequent activation of mechanosensitive channels, probably MSL10. Mechanosensitive channels contribute more to the induction of burn- and wound-triggered VPs, which is related to more abrupt pressure changes due to instantaneous stimulation, whereas upon gradual heating, prolonged stimulation causes less abrupt pressure changes. In turn, ROS activate ligand-gated Ca^2+^ channels, either the proposed ROS-activated Ca^2+^-permeable plasma membrane channel, the molecular identity of which remains unknown, or GLR3.3 and GLR3.6 through the MSL10-dependent regulation of their activity. Increased cytosolic Ca^2+^ levels induce H^+^-ATPase inactivation and, as a result, VP generation. In the cases of heating and burning, additional ROS production is mediated by the Ca^2+^- or ROS-dependent activation of NADPH oxidases, mainly RBOHD. Other factors, such as heat-driven water expansion and transpiration changes upon heating, may also contribute to the mechanism of stimulus-specific VP induction.

## 4. Materials and Methods

### 4.1. Plant Material and Growth Conditions

Wheat (*Triticum aestivum* L.) cv. Daria was grown in a growth room at 24 °C under long-day conditions (16 h/8 h light/dark). For all experiments, 14–21-day-old plants, grown in pots with sand as soil, were used. Due to the low penetration of inhibitors through the wheat leaf epidermis, experiments with inhibitors were carried out using a detached wheat leaf (second mature leaf 17 cm long) cut from wheat plants and adapted in a standard solution (1 mM KCl, 0.5 mM CaCl_2_, 0.1 mM NaCl) for 17 h.

### 4.2. Local Stimulation

Local stimulation was applied to the tip (~1 cm long) of a second mature wheat leaf. Three types of stimuli were used: (1) gradual heating in a cuvette with water to 60 °C for 5–7 min; (2) burning with a flame for 3 s; (3) mechanical wounding by crushing with a plastic cylinder. One single stimulation experiment was carried out per plant. Before stimulation, wheat plants were removed from the growth room and acclimated for a minimum of 1 h in the recording room at ~24 °C.

### 4.3. Extracellular Measurements of Electrical Signals

Surface potentials were recorded using Ag^+^/AgCl macroelectrodes EVL-1M3 (Gomel Plant of Measuring Devices, Gomel, Belarus) filled with 3 M KCl, a high-impedance three-channel amplifier IPL-113 (Semico, Novosibirsk, Russia) and a personal computer. Three measuring electrodes were placed on the second mature wheat leaf with an inter-electrode distance of 3 cm and a distance of 3 cm between the damage site and the first electrode. The interface between the measuring electrode and the leaf was a cotton thread wetted with a standard solution. A reference electrode was placed in the soil during recordings from whole plants or in a standard solution surrounding the leaf cut during recordings from detached leaves. Surface potential recordings were acquired at 1 Hz.

### 4.4. Inhibitor Studies

To investigate the features of the mechanisms of generation and propagation of VP induced by different stimuli, inhibitor analysis was performed using the plasma membrane H^+^-ATPase inhibitor sodium orthovanadate (2 mM), the plasma membrane Ca^2+^-permeable channel blocker lanthanum chloride (5 mM), the mechanosensitive channel inhibitor gadolinium chloride (10 mM), the scavenger of ROS DMTU (1 mM) [23,24], the inhibitor of NADPH oxidases DPI (20 μM) [25,26], the cell wall peroxidase inhibitor and the mitochondrial alternative oxidase inhibitor SHAM (1 mM) [27,28]. All chemicals were from Sigma-Aldrich (St. Louis, MO, USA). Solutions for chemical treatments were made in a standard solution. The solutions were loaded into a detached wheat leaf by vacuum infiltration. To do this, the cut of the detached leaf was immersed in a solution of the corresponding compound, and then exposed to one cycle of vacuum infiltration for 5 min at 70 kPa in a vacuum desiccator. Control experiments were carried out in exactly the same way via infiltration with a standard solution. All experiments were carried out 1.5 h after infiltration.

### 4.5. Monitoring of Leaf Thickness

Changes in wheat leaf thickness were monitored to detect hydraulic signals. The leaf thickness was measured using a system including an OL1 optical micrometer (SICK, Düsseldorf, Germany), an AOD1 evaluation unit (SICK, Düsseldorf, Germany) and LTR12 analog-to-digital converters in the LTR-EU-2-5 crate (L-Card, Moscow, Russia). Changes in leaf thickness were recorded at 10 Hz using L-Card Measurement Studio software (version 1.1.0) (L-Card, Moscow, Russia). A wheat leaf was fixed at an equal distance between the sender unit and the receiver unit so that the shading of the light band by the leaf, recorded by the receiver unit, corresponded to the leaf thickness. Regions of interest (3 mm) were located at distances of 4.5, 7.5 and 10.5 cm from the stimulation area (Figure 1). Simultaneously with the monitoring of leaf thickness, surface electrical potentials were recorded.

### 4.6. Measurements of Leaf Stomatal Conductance

A GFS-3000 gas analyzer (Heinz Walz GmbH, Effeltrich, Germany) and Dual-PAM gas-exchange Cuvette 3010-Dual common measuring head (Heinz Walz GmbH, Effeltrich, Germany) were used for investigations of leaf stomatal conductance, which was automatically calculated by GFS-Win software (version 3.82) (Heinz Walz GmbH, Effeltrich, Germany). The CO_2_ concentration in the measuring cuvette was 360 ppm, the relative humidity was about 70%, and the temperature was 23 °C. Blue actinic light (460 nm, 240 µmol m^−2^ s^−1^) were used in the experiments. The measuring cuvette was placed on the second mature wheat leaf at a distance of 4.5 cm from the stimulation area. Leaf stomatal conductance recordings were acquired at 1 Hz.

### 4.7. Statistical Analysis

All experiments with each stimulus type were repeated with at least 10 independent biological replicates. Each replicate was performed on a separate wheat plant. Results are represented as means ± SEM, first-order derivative, typical records of individual measurements. Statistical significance in pair-wise comparisons was evaluated by Student’s *t*-test. For multiple comparisons, two-way ANOVA were performed. The level of statistical significance was set at *p* < 0.05. Statistical analysis was performed using Microsoft Excel (version 2409) and GraphPad Prism 6 software (version 6.07) (GraphPad Software, Boston, MA, USA).

## 5. Conclusions

Thus, the study revealed differences in the parameters of VPs induced by different local stimuli: less of a decrement upon heating compared to burning and wounding, and the smallest amplitude upon wounding. Differences in VP parameters indicate the possibility of the VP-mediated induction of stimulus-specific systemic functional responses [1,2]. These findings suggest that the VP is not only a signal with information about the damaging effect, but also has the potential to encode information about the stimulus. The mechanism of the VP-mediated induction of systemic responses is most likely based on shifts in the concentrations of ions, such as Ca^2+^ and H^+^, accompanying VP generation [1,2]. Taken together, these findings suggest that the stimulus specificity of VP originates in the features of the chemical and hydraulic signals that form the VP, which, in turn, can influence changes in ion concentrations.

However, there are outstanding questions about the mechanisms of VP formation and their roles in the regulation of the physiological state of plants. Can hydraulic signals independently perform regulatory functions in plants? What is the molecular identity of ROS-activated Ca^2+^-permeable channels? What is the precise molecular pathway for VP generation? How do aquaporins play a role in generating VP and hydraulic signals? These and many other questions require further research.

Answers to these questions will facilitate the practical implementation of the acquired knowledge about the mechanisms of specificity of electrical signals in plants, primarily for solving agricultural problems. One of the priority applications is the identification of critical stressors based on the monitoring of electrical activity. To date, there are more works aimed at identifying the influencing stressor based on the classification of plant electrical signals using machine learning methods [83,84,85], including herbivore [86] and pathogen attacks [87], early detection of water [88,89] and nitrogen deficiency [90], etc. Another problem is to assess the resistance of plants based on the characteristics of electrical reactions under the influence of various stressors, such as salinity [91], high temperatures [91] and heavy metals [92], which allows the selection of the most resistant varieties at the early stages of selection. Other problems may also be addressed, including the modification of resistance to stressors by regulating the electrical activity of plants.

## Data Availability

The data that support the findings of this study are available from the corresponding author upon reasonable request.

## Supplementary Materials

The following supporting information can be downloaded at: https://www.mdpi.com/article/10.3390/plants13202896/s1, Figure S1: Dependences of the amplitude of leaf thickness reduction on the distance to the area of local stimulation induced by local heating, burning or wounding in wheat plants.
