# Supplementary material for: Analysis of the Mechanisms Underlying the Specificity of the Variation Potential Induced by Different Stimuli
Source: Plants (Basel). 2024 Oct 16;13(20):2896. doi: 10.3390/plants13202896 (PMC11511009; doi:10.3390/plants13202896)
Supplement: Supplementary file 1 [file plants-13-02896-s001.zip › plants-3242101-Figure S1.pdf]

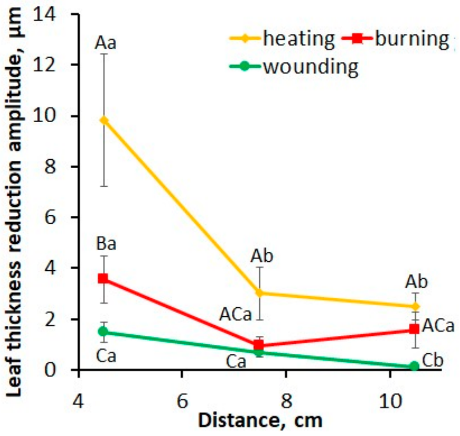

Figure S1. Dependence of the amplitude of leaf thickness reduction on the distance to the area of local stimulation upon heating, burning or wounding in wheat plants. Data are means ± SEM. Different uppercase letters indicate statistically significant differences between stimuli, different lowercase letters indicate statistically significant differences between distances within a single stimulus ($P < 0.05$).
